# Assessing the Feasibility of Using Kinect 3D Images to Predict Light Lamb Carcasses Composition from Leg Volume

**DOI:** 10.3390/ani11123595

**Published:** 2021-12-19

**Authors:** Severiano R. Silva, Mariana Almeida, Isabella Condotta, André Arantes, Cristina Guedes, Virgínia Santos

**Affiliations:** 1 Veterinary and Animal Research Centre (CECAV), Associate Laboratory of Animal and Veterinary Science (AL4AnimalS), University of Trás-os-Montes e Alto Douro, Quinta de Prados, 5000-801 Vila Real, Portugal; ssilva@utad.pt (S.R.S.); cguedes@utad.pt (C.G.); vsantos@utad.pt (V.S.); 2 Animal Science Department, University of Trás-os-Montes e Alto Douro, Quinta de Prados, 5000-801 Vila Real, Portugal; arantex@hotmail.com; 3 Department of Animal Sciences, College of Agricultural, Consumer and Environmental Sciences, University of Illinois at Urbana-Champaign, Urbana, IL 61801, USA; icfsc@illinois.edu

**Keywords:** Microsoft Kinect, lambs, carcass composition, leg volume, 3D image

## Abstract

**Simple Summary:**

The present study aimed to evaluate the accuracy of the leg volume obtained by the Microsoft Kinect sensor to predict the carcass composition of twenty-two male light lambs. The carcasses were divided into eight cuts, which were grouped according to their commercial value into high-value, medium value, and low-value. Several linear, area, and volume leg measurements were performed to predict cut and carcass composition. The leg volume determined by 3D image reconstruction using Microsoft Kinect sensor and Archimedes principle shows the higher correlations values with cuts and carcass. Additionally, it was observed that the models, which include the leg volume obtained by the Kinect sensor, are very good in predicting the weight of the medium value and leg cuts (R^2^ of 0.763 and 0.829, respectively). Thus, the results of this study confirm the good ability to estimate cuts and body traits from light lambs with volume measurements, particularly those obtained with the Kinect 3D sensor.

**Abstract:**

This study aimed to evaluate the accuracy of the leg volume obtained by the Microsoft Kinect sensor to predict the composition of light lamb carcasses. The trial was performed on carcasses of twenty-two male lambs (17.6 ± 1.8 kg, body weight). The carcasses were split into eight cuts, divided into three groups according to their commercial value: high-value, medium value, and low-value group. Linear, area, and volume of leg measurements were obtained to predict carcass and cuts composition. The leg volume was acquired by two different methodologies: 3D image reconstruction using a Microsoft Kinect sensor and Archimedes principle. The correlation between these two leg measurements was significant (r = 0.815, *p* < 0.01). The models to predict cuts and carcass traits that include leg Kinect 3D sensor volume are very good in predicting the weight of the medium value and leg cuts (R^2^ of 0.763 and 0.829, respectively). Furthermore, the model, which includes the Kinect leg volume, explained 85% of its variation for the carcass muscle. The results of this study confirm the good ability to estimate cuts and carcass traits of light lamb carcasses with leg volume obtained with the Kinect 3D sensor.

## 1. Introduction

The evaluation of carcass characteristics is a fundamental process for attributing the quality and value of the animal at slaughter. Over the last three decades, several approaches for carcass grading systems supported by objective measurements have been developed for cattle [1,2], pigs [3], and sheep [4,5]. In general, these works aimed to classify the carcass quality based on non-destructive image analysis techniques by introducing consistency, accuracy, credibility, and confidence in the value assessment of the carcass [1,6]. The ultimate aim is to replace the subjective evaluation grounded on standards and move towards advanced value-based payment systems [7]. These evaluations techniques are also relevant for the classification of light lamb carcasses produced in Mediterranean countries. In these regions the slaughter of lambs from dairy breeds at 4 to 6 week of age with a low body weight ranging approximately between 10 and 11 kg is one of the most widely used production system [8]. These light carcasses are from different local breeds and particularly those with Protect Denomination of Origin (PDO) and Protected Geographical Indication (PGI) quality labels are regarded with high edible value. “Borrego Terrincho” and “Cordeiro Mirandês” with PDO [9,10] or “Lechazo de Castilla y León” and “Ternasco de Aragón” with PGI [11] are some examples of light lamb products with quality labels that can be found in Portuguese and Spanish markets. Unlike heavier lamb carcasses for light carcass weights (carcass weight < 13 kg), there is no conformation assessment, and therefore they are tab penalized due to their naturally poor morphology [12].

Video image analysis (VIA) has been one of the most researched technologies and commercial solutions for carcass assessment of beef [1,13], pork [14], and lamb [15,16]. The VIA equipment used in these studies has been tailored for large industrial slaughterhouse plants that mainly use color and dimensional data obtained from 2D images of lateral or dorsal views of carcasses. From these images, data are extracted to estimate yield, conformation, and EUROP fat and conformation scores [3]. However, some work has been developed in recent years using 3D sensors to obtain information on carcasses [17,18] and live animals [19,20]. Some of these works use 3D sensing devices such as stereoscopic and time-of-flight cameras. Advanced depth imaging with low-cost sensors such as Microsoft Kinect is increasingly used in animal science [21,22]. The latter sensor can provide 3D data from its infrared and RGB color (Red, Green, Blue) images, representing a flexible objective technology that can be applied in predicting carcass composition, cut distribution, and lean yield prediction of carcasses. Despite the potential of this technology, there is a lack of information on its application to lamb carcasses. In this regard, this preliminary study aimed to evaluate the accuracy of leg volume obtained by the Microsoft Kinect sensor to predict the composition of light lamb carcasses.

## 2. Materials and Methods

### 2.1. Animals and Carcasses

The trial took place at the animal facilities of the University of Trás-os-Montes and Alto Douro (UTAD) at Vila Real (Portugal), and all the handling was performed according to the Portuguese law on animal welfare in experimental research. The protocol was approved by the ORBEA (Animal Welfare Body) of UTAD (669-e-DZ-2018). The trial was performed on twenty-two Churra da Terra Quente male lambs, weighing 17.6 ± 1.8 kg. After slaughter, the carcasses were obtained and then refrigerated at 4 °C for 24 h. After this period, the cold carcass weight (CCW) was recorded.

### 2.2. Leg Area and Leg Linear Carcass Measurements

Carcasses were measured in two ways. First, the perimeter measure of the hindquarter was obtained from the entire carcass. Then, the carcasses were split along the spine, and the left side was used to perform the remaining measurements. For this, the procedure using image analysis proposed by Batista et al. [23] was used. Briefly, the carcass measurements were recorded from photographic images of the left outer side. The images were obtained with a digital camera (Nikon D3100, Mitsubishi, Tóquio, Japan) with an 8-megapixel sensor positioned at 3 m from the carcasses and under a constant standard artificial light. The acquired images were analysed with the Fiji software (ImageJ 1.49u, National Institutes of Health, MA, USA) [24] to calculate the measurements of leg area, leg length, leg perimeter, hind quarter perimeter, and three widths (thinnest and largest leg width and minimum waist width).

### 2.3. Carcass Cuts and Composition

**Figure 1:**
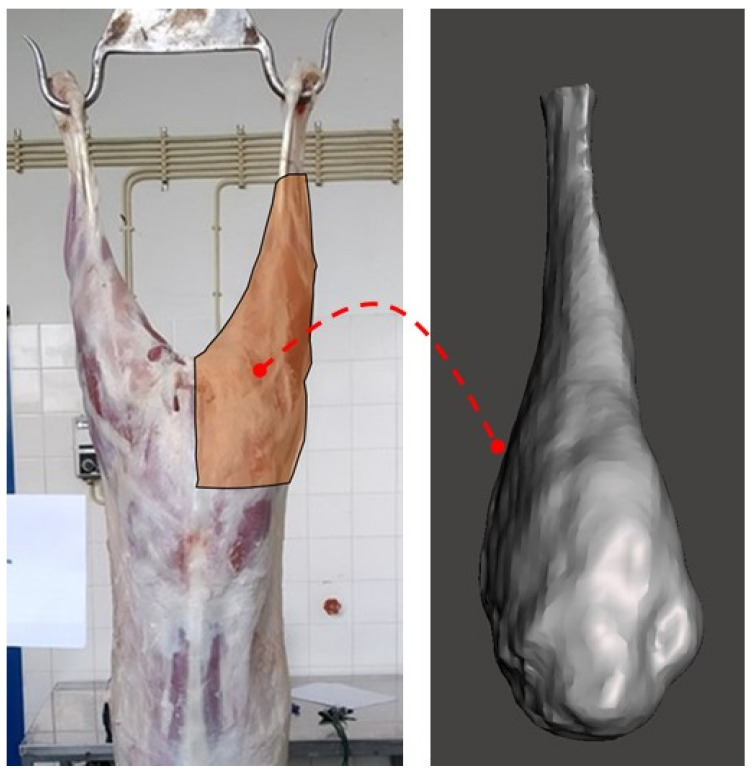
The left image illustrates the outline of the leg cut used to determine the volume either by Archimedes’ or 3D image reconstruction method. The right image shows the 3D leg model obtained with the Kinect sensor.

After obtaining carcass measurements, the half-carcasses were divided into eight cuts: neck, shoulder, breast, anterior rib, rib, loin, chump, and leg, as described by Santos et al. [25]. Following the methodology proposed by Rodrigues et al. [26], the cuts were split into three groups according to their commercial value: high-value group (HVC), which included the leg, chump, and loin; medium value group (MVC) that included rib and shoulder; and low-value group (LVC) in which the breast, anterior rib, and neck were included. The leg (Figure 1) volume was then acquired by two different methodologies: 3D image reconstruction using a Microsoft Kinect sensor and Archimedes principle. After that, all cuts were dissected into muscle, fat (which includes subcutaneous and intermuscular fats), and bone, according to the methodology proposed by Panea et al. [27]. All dissection work was performed in a room under a controlled environment.

### 2.4. Leg Volume with Kinect 3D Image

A Microsoft Kinect 2.0 sensor (Microsoft, NM, USA) was used to acquire the leg volume by 3D image reconstruction. This sensor incorporates an RGB camera and a depth infrared sensor, which is the main feature used in the study. The minimal computer hardware requirements needed for taking 3D images with the Kinect sensor are Windows 8 operational system, Dual-core 3.1GHz processor, 4GB RAM, and a 3.0 USB port. The Kinect Fusion Explorer program, included in the Windows Software Development Kit provided by Microsoft, was used to scan the leg and build the 3D image model. This software allows choosing the maximum and minimum distances from the depth sensor to the object used to capture the image. It also allows capturing the object colors and choosing how detailed the final model will be. The program configuration will vary due to differences in luminosity and reflection of the light in the surrounding environment and the scanned object. Therefore, there are no standard configurations to run the program as they will be different for different objects. Furthermore, the computer specifications also affect the image acquisition performance: the better the computer, the more detailed and smoother is the image generated.

For the 3D model generation, the cut was hung in a structure that allowed the Kinect sensor to be moved around it undisturbed. The camera was held at chest height and slowly moved around the cut, so the program had time to construct a model with the most detail possible. All the models were exported in STL format. After that, models were imported into the Autodesk Meshmixer program to determine the leg volume. For that, the two steps were performed. First, the background was segmented from the leg region using an edition tool of the Meshmixer toolbar. After that, leg measurements were acquired. For that, the manually acquired leg length, in centimeters, was input in the Z-axis of the Meshmixer toolbar to serve as a reference for px to metric units’ transformation. Then, the program automatically determined the leg volumes.

### 2.5. Leg Volume with Archimedes Principle

The Archimedes principle was also used to acquire the leg volume, as this is the standard method used to acquire that measurement. First, a 5 L container was filled with water until the superficial tension was reached. The container was large enough to fit the entire leg. This container was placed inside another container. After that, the cut was slowly inserted into the water, and the outside container captured excedent water. It was assumed that the water density value is 1 kg/L (one kilogram per liter) and that one liter of water is equivalent to 1000 cm^3^. With these assumptions and based on the weight of the spilled water, the leg volume was calculated. Special care was taken with measuring the spilled water. For this, a precision balance (Precisa LT 6200C, Precisa, Livingston, UK) with a resolution of 0.1 g was used, and all procedures for weighing the water were kept constant.

### 2.6. Statistical Analysis

A descriptive statistical analysis was performed. Mean, standard deviation, maximum and minimum value, and coefficient of variation were obtained for the weight of cold carcass, cuts, and carcass composition, and for the measurements of carcass and leg. A correlation analysis was performed to examine the relationship between cut and carcass composition and all carcass and leg measurements. Additionally, a correlation analysis was performed between the leg volumes obtained with the Archimedes principle and with the Kinect sensor. Additionally, a multiple regression analysis that included the CCW and the carcass and leg measurements was performed. The best equations were chosen based on the precision of the prediction model, measured by the coefficient of determination (R^2^), and the residual standard deviation (RSD). As an indicator of the overall prediction ability, the models were also evaluated for the ratio of prediction to deviation (RPD), which is calculated as the ratio of standard deviation (sd) values to the RSD of the multiple regression (RPD = sd/RSD). All statistical procedures were carried out using the JMP software version 15 (SAS, Cary, NC, USA) [28].

## 3. Results and Discussion

### 3.1. Cold Carcass Weight, Cuts, and Carcass Composition

Table 1 summarizes the descriptive statistics (mean, standard deviation, minimum, maximum, and coefficient of variation) for cold carcass weight, cuts, and carcass composition. The carcasses show a small weight range (CCW between 6.85 and 9.91 kg) and a reduced variation (CV = 10.1%). These characteristics were observed in other studies with light carcasses with CV between 4 and 13% [25,29,30]. As previously mentioned, light carcasses are closely associated with the traditional lamb meat production in Mediterranean countries [8,12] and are often linked to PDO and PGI quality labels, which present in their specification and narrow carcass weight ranges [13], which explains the low variation for this type of carcass. Regarding muscle and fat from cuts and carcass, as expected, the variation is higher for fat (CV between 19.5 and 34.8%) than for muscle (CV between 7.6 and 12.2%). Still, regarding fat, a more significant variation is observed in LVC than in HVC cuts (CV = 34.8 vs. 16.5%, respectively). These results are in line with what was pointed out by [31] that the cuts with the highest fat content are those included in the LVC, such as the breast, with 42.1%, and the leanest cut was the leg.

The mean, standard deviation, minimum, maximum, and coefficient of variation of leg measurements obtained through VIA are presented in Table 2.

All measurements show a reduced variation (CV between 4.2 and 9.1%), which agrees with the reduced variation of CCW of the studied carcasses.

### 3.2. Correlation between Measurements and Composition of Cut and Carcass

Table 3 shows the correlation values of the leg measurements with cut and carcass weight and composition traits. In general, there is a significant correlation between the different leg measurements and cut and carcass traits. However, only the leg volume measurements were significantly correlated with all cuts and carcass traits (r between 0.417, *p* < 0.05 and 0.835, *p* < 0.01). Additionally, the correlation values of leg volume (Archimedes and Kinect 3D) with cuts and carcass traits show a very similar pattern, which reflects the relationship between these two leg measurements (r = 0.815, *p* < 0.01). The leg length is the measurement that shows the smallest correlations with cuts and carcass traits (r between 0.084, *p* > 0.05 and 0.450, *p* < 0.05). The leg area measurement presents intermediate correlation values with only two non-significant correlations (r = 0.337 and 0.413, *p* > 0.05 for leg muscle and LVC muscle, respectively). Additionally, the correlation values are generally less significant with the fat trait, whereas the correlation values are comparable for the cut and carcass weight and muscle. The value of the measurements obtained in the leg, in general, have been observed by other authors who have used linear and area measurements to predict cuts and lean meat variation of carcasses [23]. In this work, which studied light carcass, although there is no simple correlation between the measurements obtained in the carcass and the cuts, it is possible to observe that area and perimeter of the leg are included on the HVC, MVC, and LVC cut weight prediction models, whereas for models’ prediction of lean meat weight always include leg area measurements.

Multiple regressions were studied with each of the leg measurements and the CCW. The equations that best explain the weight, muscle, and fat composition of the cuts and carcass are presented in Table 4. Carcass weight is extensively used in studies to predict carcass composition as it is an accessible variable and shows to be an informative predictor for primal cut variations [32,33].

The leg volume measurements are the most used in multiple regressions with CCW to estimate cuts and carcass traits. Of the fifteen models presented, only those estimating LVC and fat carcass did not include leg volume measurements. It is also observed that for the leg volume measurements, the one obtained by the Kinect 3D sensor was the most used (9 out of 13 models). The models that include leg volume measurements obtained by the Kinect sensor are shown to be very good in predicting the weight of the MVC and leg cuts (R^2^ of 0.763 and 0.829, RDP of 2.0 and 2.3, respectively). In turn, for the HVC, the model that includes the Archimedes volume measurement and the CCW presents a very good prediction (R^2^ = 0.817, RDP = 2.2). These values are very close to what was observed for the model that included the leg volume measurement obtained by the Kinect sensor (R^2^ = 0.804, RDP = 2.2; data not shown), which reinforces its ability to predict cut traits. The results of cuts muscle estimation models are more modest, and only the MVC muscle estimation using the leg volume measurement with Kinect 3D has good prediction capacity (R^2^ = 0.723, RDP = 1.8). For the carcass muscle estimation model with the Kinect leg volume measurement, it was possible to explain 85% of its variation with the model classified as very good (RPD = 2.4). Regarding the cuts of fat, all models were classified as poor or fair (R^2^ between 0.433 and 0.577, RPD between 1.3 and 1.5). In turn, for the carcass fat estimate, the model showed higher capacity but with the leg area measurement included in the model (R^2^ = 0.742; RDP = 1.9).

Two-dimensional video image analysis is one such technique that has been used for carcass evaluation of different species. Although 2D shape information can be useful, 3D information is preferable to ensure more accurate weight estimates [35] and, therefore, better carcass quality prediction. There is a growing interest in developing prediction models of carcass and meat quality traits using 3D measurements, for example, computed tomography (CT) and other image-based approaches [5,7]. With CT, most works target pigs [36,37]. This priority is understandable for the economic expression of this species. Despite this, contributions to predicting the body and carcass composition of small ruminants were also made with CT using 3D measurements [38,39]. In general, the results are promising, and it is expected that with the equipment progress and with advances in 3D carcass modelling software, it will be possible to speed up all procedures to obtain accurate information based on 3D images both in vivo and in the carcass [39]. In addition to CT, other techniques are also evolving to obtain 3D images, such as dual-energy X-ray absorptiometry—DXA [40]. Despite the enormous value of CT and DXA, its cost and complexity of use are substantial limitations as an aid to predict carcass traits that would maximize carcass value. However, the potential of three-dimensional (3D) image reconstruction has not yet been largely explored, especially for lamb carcass assessment.

The low-cost Microsoft Kinect depth sensor has been used as a tool for rapid, reliable, objective, and non-invasive measurements in animal science [12], demonstrating potential as a carcass measurement device. Therefore, the present work evaluated the feasibility of using the Microsoft Kinect sensor to obtain the volume (3D reconstruction) of twenty-two light lamb legs. The volumes obtained were then assessed for their significance in predictive models of carcass and cuts traits. Nine out of fifteen models analyzed required the use of leg volume obtained through 3D reconstruction, along with cold carcass weight, as an independent variable, with the majority of the models presenting an R^2^ of 0.7 or higher. Such positive results expose the potential of using a Kinect sensor to predict light lamb carcass composition. However, some constraints with the proposed method must be overcome to take full advantage of the Kinect sensor capability in estimating carcass traits. The constraints rely on reduced variables, variation and sample size, limiting predictive capabilities. Furthermore, the method is not fully automated, requiring a careful scan of the cut by manually moving the sensor and the manual input of the leg length for unit calibration. Therefore, the possibility of obtaining leg volume measurements automatically is an attribute that must be followed to improve the capacity of this technique. In this way, it will be possible to make the image capture and analysis procedure faster, and in this way to overcome time as a constraint even in its potential application to breeds that give rise to carcasses with a quality label, not posing the critical problems in the chain speed of a large commercial abattoir [41] as this type of animal is generally linked to local slaughterhouses [42].

## 4. Conclusions

This study confirms the feasibility of the Kinect 3D images to predict light lamb carcasses composition from leg volume. Accordingly, the following steps of this research should include larger sample size and focus on the automation of both acquisition and analysis of 3D images in order to produce a more reliable, fast, and practical method of lamb light carcass assessment; and with light carcasses from different breeds and using the Kinect 3D as a tool to find a benchmark for quality of that type of carcass.

## Figures and Tables

**Table 1:** Mean, standard deviation (sd), minimum, maximum, and coefficient of variation (CV) of cold carcass weight, cuts, and carcass composition.

Traits	Mean	sd	Min	Max	CV (%)
Cold carcass weight (kg)	8.66	0.88	6.85	9.91	10.1
Cut					
Leg (g)	1145.27	82.39	973.80	1243.00	7.2
Leg muscle (g)	698.41	53.13	566.90	772.70	7.6
Leg fat (g)	113.37	23.26	80.10	153.80	20.5
HVC (g)	1818.93	201.66	1447.40	2127.30	11.1
HVC muscle (g)	1063.09	129.76	826.10	1292.30	12.2
HCV fat (g)	235.30	45.80	154.40	308.60	19.5
MVC (g)	1082.74	111.29	840.50	1216.50	10.3
MVC muscle (g)	583.38	71.33	439.40	700.60	12.2
MCV fat (g)	127.32	34.78	84.50	202.70	27.3
LVC (g)	1056.58	119.00	797.80	1274.80	11.3
LVC muscle (g)	489.11	48.92	406.10	572.00	10.0
LCV fat (g)	181.35	63.04	75.50	289.00	34.8
Carcass* (g)	3958.24	376.02	3088.80	4447.50	9.5
Carcass muscle (g)	2135.58	222.49	1724.70	2463.10	10.4
Carcass fat (g)	543.97	129.48	323.60	698.90	23.8

HVC = high value cuts; MVC = medium value cuts; LVC = low value cuts; Carcass* = sum of HVC, MVC and LVC

**Table 2:** Mean, standard deviation (sd), minimum, maximum, and coefficient of variation (CV) of the leg measurements.

Leg Measurements		Mean	sd	Min	Max	CV (%)
Length (cm)		28.50	1.99	25.00	32.00	7.0
Width (cm)	Thinnest width of leg (LW1)	12.62	0.96	10.90	14.00	7.6
	Largest width of the leg (LW2)	13.51	0.71	12.00	14.50	5.3
	Minimum waist width (LW3)	13.29	0.65	11.80	14.30	4.9
Perimeter (cm)	Hind quarter	49.85	2.09	46.00	54.00	4.2
	Leg	34.41	2.00	31.00	38.00	5.8
Area (cm^2^)		367.03	26.25	324.40	412.40	7.2
Volume (cm^3^)	Archimedes (cm^3^)	1025.52	69.12	891.70	1126.10	6.7
	Kinect 3D image (cm^3^)	1036.53	94.29	865.77	1191.07	9.1

**Table 3:** Correlations between measurements and composition of cuts and carcass.

Traits	Length (cm)	Width (cm)			Perimeter (cm)		Area (cm^2^)	Volume (cm^3^)	
		LW1	LW2	LW3	Hind Quarter	Leg		Archimedes	Kinect 3D
Leg (g)	0.433 *	0.393	0.537 *	0.309	0.622 **	0.486 *	0.602 **	0.807 **	0.822 **
Leg muscle (g)	0.322	0.323	0.249	0.040	0.489 *	0.182	0.337	0.762 **	0.688 **
Leg fat (g)	0.180	0.428 *	0.537 *	0.433 *	0.397	0.509 *	0.574 **	0.500 *	0.603 **
HVC (g)	0.393	0.583 **	0.687 **	0.458 *	0.482 *	0.500 *	0.736 **	0.742 **	0.727 **
HVC muscle (g)	0.438 *	0.655 **	0.624 **	0.406	0.468 *	0.351	0.708 **	0.752 **	0.659 **
HCV fat (g)	0.084	0.417	0.572 **	0.432 *	0.375	0.610 **	0.498 *	0.556 **	0.724 **
MVC (g)	0.450 *	0.589 **	0.660 **	0.531 *	0.715 **	0.290	0.700 **	0.835 **	0.736 **
MVC muscle (g)	0.294	0.456 *	0.573 **	0.435 *	0.586 **	0.371	0.633 **	0.640 **	0.714 **
MCV fat (g)	0.322	0.634 **	0.611 **	0.538 **	0.436 *	0.079	0.626 **	0.683 **	0.417 *
LVC (g)	0.309	0.308	0.438 *	0.362	0.580 **	0.189	0.445 *	0.495 *	0.529 *
LVC muscle (g)	0.379	0.243	0.415	0.156	0.512 *	0.353	0.413	0.524 *	0.716 **
LCV fat (g)	0.149	0.612 **	0.667 **	0.635 **	0.438 *	0.499 *	0.761 **	0.540 **	0.650 **
Carcass* (g)	0.441 *	0.577 **	0.686 **	0.521 *	0.701 **	0.412	0.758 **	0.822 **	0.793 **
Carcass muscle (g)	0.383	0.529 *	0.620 **	0.401	0.537 *	0.540 **	0.655 **	0.723 **	0.812 **
Carcass fat (g)	0.216	0.668 **	0.712 **	0.691 **	0.545 **	0.411	0.835 **	0.674 **	0.633 **

HVC—high value cuts; MVC—medium value cuts; LVC—low value cuts; LW1—thinnest width of leg; LW2—largest width of the leg; LW3—minimum waist width; * *p* < 0.05; ** *p* < 0.01; Correlations values without asterisk are non-significant *p* > 0.05; Carcass* = sum of HVC, MVC and LVC.

**Table 4:** The best multiple regressions for cuts and carcass traits (dependent variables) with CCW and one measurement (independent variable).

Dependent	Intercept	Independent			R^2^	RSD	RDP	*p* Value
		*X1* (CCW, kg)	*X2*					
Leg (g)	310.668	57.846	0.323	Kinect 3D (cm^3^)	0.829	35.8	2.3	<0.0001
Leg muscle (g)	102.564	5.359	0.536	Archimedes (cm^3^)	0.585	36.0	1.5	0.0002
Leg fat (g)	−58.596	10.983	0.074	Kinect 3D (cm^3^)	0.433	18.4	1.3	0.0046
HVC (g)	−235.776	176.173	0.516	Archimedes (cm^3^)	0.817	90.8	2.2	<0.0001
HVC muscle (g)	−310.7	78.111	0.680	Archimedes (cm^3^)	0.692	75.8	1.7	<0.0001
HCV fat (g)	−162.143	14.349	0.265	Kinect 3D (cm^3^)	0.555	32.1	1.4	0.0005
MVC (g)	53.96	93.696	0.211	Kinect 3D (cm^3^)	0.763	57.0	2.0	<0.0001
MVC muscle (g)	−57.132	58.871	0.127	Kinect 3D (cm^3^)	0.723	39.5	1.8	<0.0001
MCV fat (g)	−217.263	8.248	0.266	Archimedes (cm^3^)	0.486	26.2	1.3	0.0018
LVC (g)	−499.473	19.743	27.785	Perimeter hind quarter	0.349	100.9	1.2	0.017
LVC muscle (g)	78.175	4.55	0.36	Kinect 3D (cm^3^)	0.515	35.8	1.4	0.001
LCV fat (g)	−331.271	44.41	0.124	Kinect 3D (cm^3^)	0.577	43.1	1.5	0.0003
Carcass* (g)	235.941	313.033	0.98	Kinect 3D (cm^3^)	0.845	155.7	2.4	<0.0001
Carcass muscle (g)	−105.423	171.687	0.731	Kinect 3D (cm^3^)	0.845	92.0	2.4	<0.0001
Carcass fat (g)	−883.099	56.078	2.565	Area leg (cm^2^)	0.742	69.2	1.9	<0.0001

HVC—high-value cuts; MVC—medium value cuts; LVC—low-value cuts; CCW—cold carcass weight; R^2^—coefficient of determination; RSD—residual standard deviation; RPD—ratio of prediction to deviation. RPD < 1.0 indicates very poor model/predictions; RPD between 1.0 and 1.4 indicates poor model/predictions; RPD between 1.4 and 1.8 indicates fair model/predictions; RPD values between 1.8 and 2.0 indicates good model/predictions; RPD between 2.0 and 2.5 indicates very good, quantitative model/predictions, and RPD > 2.5 indicates excellent model/predictions [34]; Carcass* = sum of HVC, MVC and LVC.

## Data Availability

The data presented in this study are available on request from the corresponding author. The data are not publicly available due to privacy reasons.
